# Prevalence and risk factors of keratoconus (including oxidative stress biomarkers) in a cohort study of Shiraz university of medical science employees in Iran

**DOI:** 10.1186/s12886-023-02934-0

**Published:** 2023-04-28

**Authors:** Sahar Mohaghegh, Haleh Kangari, Seyed Jalil Masoumi, Shahram Bamdad, Saeed Rahmani, Saeed Abdi, Nagham Fazil, Saeedeh Shahbazi

**Affiliations:** 1 Department of Optometry, School of Rehabilitation, Shahid Beheshti University of Medical Sciences, Tehran, Iran; 2 Nutrition Research Center, Department of Clinical Nutrition, School of Nutrition and Food Sciences, Shiraz University of Medical Science, Shiraz, Iran; 3 Poostchi Ophthalmology Research Center, Department of Ophthalmology, School of Medicine, Shiraz University of Medical Sciences, Shiraz, Iran; 4 Labbafinejad Hospital, Shahid Beheshti University of Medical Sciences and health services, Tehran, Iran

**Keywords:** Keratoconus, LDL, HDL, BMI, Oxidative stress, Low density lipoprotein cholesterols, Triglyceride, Body mass index, Inflammation

## Abstract

**Background:**

To determine the prevalence of keratoconus in Shiraz University of Medical Sciences Employees and the related risk factors including oxidative stress biomarkers.

**Methods:**

2546 subjects’ mean age ± SD, 40.35 ± 6.70 (46% male) were recruited. All participants underwent objective refraction using auto-refractometer and retinoscopy, followed by subjective refraction, and bio-microscopy. Pentacam imaging was performed for the detected keratoconus patients. The prevalence of keratoconus and frequency of the visual impairment among keratoconus cases were evaluated. Potential risk factors of sex, age, family history of keratoconus, body mass index ≥ 30 kg/m^2^, serum levels of glucose ≥ 100 mg/d, low-density-lipoprotein-cholesterol (LDL) ≥ 110 mg/dL, high-density-lipoprotein-cholesterol ≤ 40 mg/d, and triglycerides ≥ 150 mg/dL in the blood were evaluated.

**Results:**

The prevalence of keratoconus at least in one eye was 0.98% (95% CI: 0.6- 1.4%). The best corrected visual acuity in the keratoconus group was 0.06 ± 0.1 and the rest of the population was 0.01 ± 0.07 logMAR (p < 0.001). The frequency of visual impairment in the keratoconus group was zero. Odds ratios of the family history of keratoconus (21.00, 95% CI: 9.00–48.00, p < 0.001) and LDL ≥ 110 mg/dL (3.00, 95% CI: 1.20–6.40, p = 0.01) were significant.

**Conclusions:**

Keratoconus is rare and is not considered a risk factor for visual impairment. A family history of keratoconus and elevated serum LDL levels are contributing risk factors, suggesting an inflammatory background for the disease. Serum levels of LDL ≥ 110 mg/dL in the blood increased the risk of keratoconus three folds.

## Background

Keratoconus (KCN) is a bilateral progressive corneal ectasia characterized by corneal steepening, irregular astigmatism, and myopia that results in decreased visual acuity that cannot be corrected with spectacle in moderate-to-advanced cases [1, 2]. The global prevalence of KCN has been reported to be 1.40 per 1000 population based on a systematic review study [3]. The prevalence and incidence of KCN vary according to ethnicity and geographical location. The prevalence of KCN has been reported as low as 4 per 1000 in Denmark [4] and up to 40 per 1000 in the rural area of Iran [5]. Differences in the prevalence of KCN are related to differences in race, environmental factors, and measurement methods [6]. Decreased visual acuity and early onset and chronic disease features affect the patient’s quality of life and impose significant social and economic burdens [7]. KCN is a complex disease, and its underlying causes are still under investigation. The pathogenesis of KCN involves a combination of genetic and environmental factors [8]. Some risk factors, such as family history [9, 10], eye rubbing [3], and inflammatory conditions [11–13], have been revealed in previous studies. Evidence suggests that KCN is associated with oxidative stress [14]. Oxidative damage induced by reactive oxygen species [15]. Some known factors and biomarkers are related to oxidative stress in metabolic diseases, such as high body mass index (BMI) [16], hyperglycemia [17], high levels of low-density lipoprotein (LDL) [18, 19], low levels of high-density lipoprotein (HDL) [20], and high levels of triglyceride (TG) [21]. As oxidative stress was considered a risk factor for KCN, these factors may be associated with KCN. Previous investigation revealed some associations between KCN and lipid profiles [12, 13, 22]. A pilot molecular study revealed differences between metabolic pathways involved in energy production, lipid metabolism, and amino acid metabolism between KCN and normal corneas [22]. Elevated levels of inflammatory biomarkers, including monocyte-to-HDL-cholesterol ratio and neutrophil-to-lymphocyte ratio, in KCNs have been reported [13]. Therefore, the evaluation of lipid profiles in KCNs may shed light into pathogenesis of the disease.

Considering genetic and environmental differences in various populations, we aimed to investigate the prevalence of KCN among the Shiraz University of Medical Science employees in the current study. Since a complete lipid profile study in KCNs has not been evaluated before, in the current study, we aim to investigate the association between KCN and potential risk factors of age, sex, family history of KCN, education level, high BMI, hyperglycemia, elevated serum levels of LDL and TG, and Low serum levels of HDL in the blood.

## Methods

A total of 2546 Shiraz University of Medical Science employees, aged 21 to 62 years, were recruited in the current study. Data were gathered from 2019 to 2020 at Shiraz University of Medical Sciences. Employees were invited to participate in the university cohort study clinic equipped for this purpose. All participants who agreed to participate in the study were informed of the study’s objectives and the examination methods and written informed consent was obtained from them. In Iran, the Ministry of Health and Medical Education launched a nationwide cohort study-Prospective Epidemiological Research Studies in Iran (PERSIAN)- to identify the most prevalent noncommunicable diseases among Iran’s ethnic groups and to investigate effective methods of prevention. The PERSIAN study consists of 4 population-based cohorts; the adult component (the PERSIAN Cohort Study), is a prospective cohort study including 180,000 persons aged 35–70 years from 18 distinct areas of Iran [23]. Shiraz University of Medical Science (SUMS) Employees’ Health cohort study is a part of PERSIAN cohort study, from which we extracted our data. The study protocol was conducted under the tenets of the Declaration of Helsinki and was approved by the ethics committee (IR.SBMU.RETECH.REC.1400.713). A digital code was generated for each participant as an identification number, and the confidentiality of the data was confirmed. The participants were asked to fast for a blood test. After taking the blood test, participants had breakfast, and the rest of the measurements were performed. Demographic information, including age, sex, education level, height (cm), and weight (kg), was recorded for all participants). Height and weight were measured with a digital height and meter measurement device. The BMI was calculated using the following formula: BMI = weight (kg)/ height^2^ (m^2^). For studies in non-East Asians, BMI categories were defined as lean < 25, overweight 25-29.9, and obese ≥ 30 kg/m2. In the current study we used the cut off of BMI ≥ 30 kg/m^2^ as a high BMI [24].

## Ocular examination

A comprehensive ocular examination, including objective refraction with an auto refractometer (rm 800, Topcon, Tokyo, Japan) followed by retinoscopy and subjective refraction, bio-microscopy, intraocular pressure measurements with non-contact tonometry (Topcon CT 80; Tokyo, Japan), and dilated fundus examination was performed for all of the participants. The case history and self-claimed family history of ocular conditions concerning KCN disease were recorded for all of the participants. Uncorrected and best-corrected visual acuity (UCVA and BCVA) was obtained using the logarithm of the minimum angle of resolution (logMAR) chart. Best corrected visual acuity worse than 0.5 LogMAR was considered as visual impairment according to the world health organization definition [25].

### Keratoconus diagnosis

The primary KCN diagnosis was obtained by the sign of scissor reflex and irregular astigmatism in retinoscopy or the signs of Vogt’s striae, Fleischer rings, and Munson’s sign in bio-microscopy [6]. The diagnosis of KCN was approved using Pentacam imaging (Pentacam-Hr, Oculus, Wetzlar, Germany)-for all of the suspected cases. KCN diagnosis was based on the topographic parameters, including zonal maximum keratometry in a 3-mm zone around the steepest point (zonal Kmax-3 mm), Ambrósio Relational Thickness (ART-max), inferior-superior (IS)-value, Belin-Ambrósio deviation index (BAD-D), minimum corneal thickness, and posterior elevation map. Zonal Kmax-3 mm > 48 D [26], ART-max < 339 [27], (IS)-value > 1.4 [28], BAD-D > 1.6 [29, 30] were used to confirm KCN. The final definition of KCN was established when there were clinical signs and two or more abnormal Pentacam parameters.

### Blood sampling

A regular blood test was performed with needle injection, collecting 30 mL of the blood sample. After the laboratory analysis, all blood test results were recorded for each participant. In the current study, the results factors of serum levels of fasting blood sugar, LDL, HDL, and TG mg/dL were selected for analysis. Hyperglycemia, elevated LDL, low HDL, and elevated TG levels were defined as serum levels of glucose ≥ 100 mg/dL [31], LDL ≥ 110 mg/dL, HDL ≤ 40 mg/dL, and TG ≥ 150 mg/dL, respectively in the blood according to the laboratory range [32].

### Statistical analysis

Statistical analysis was performed using SPSS version 25 (Chicago, Armonk, NY, USA). Descriptive statistics were used to describe the data features. The frequency of KCN cases was evaluated in all participants, and the frequency of visual impairment in the KCN group was also assessed. The non-parametric Mann-Withney test was used to compare KCN and non-KCN individuals. Univariate regression analysis was performed to determine the association between KCN and the potential risk factors. Possible risk factors with relevant P-values less than 0.2 were included in a multivariable logistic regression analysis. A P-value less than 0.05 was considered statistically significant.

## Results

**Fig. 1:**
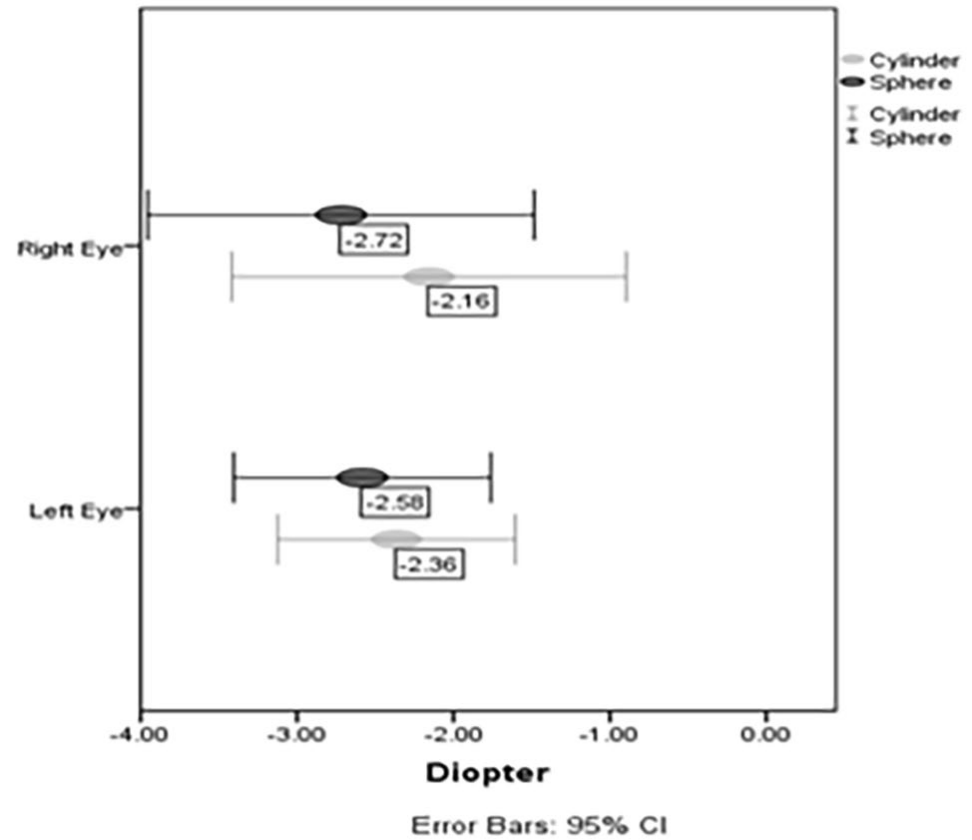
Distributions of refractive components in the keratoconus subjects’ right and left eyes

Demographic data, including age, sex, and education level, are provided in Table 1. A population of 2546 individuals, 46% male and 54% female (mean age ± SD = 40.35 ± 6.70 range 25–62 years old), were included in the analysis. 60% of the studied population had post-graduates, 30% had licenses, and 10% had diplomas. Twenty-four cases (0.98%) (95% CI: 0.6- 1.4%) were diagnosed with KCN at least in one eye, consisting of 12 males (1% in the male population) and 12 females (0.94% in the female population). The difference in the prevalence of KCN between the female and male populations was not significant (p = 0.58). The mean age + SD in KCN subjects was 39.32 ± 6.35, and in non-KCN subjects was 40.36 ± 6.71 (p = 0.43). The best corrected visual acuity in KCN subjects was 0.06 ± 0.1 logMAR, and in non-KCN subjects was 0.01 ± 0.07 logMAR (p < 0.001). The spherical equivalent in KCN subjects was − 4.00 ± 3.10, and in non-KCN subjects was − 0.70 ± 1.50 (p < 0.001). Twenty-one cases showed bilateral KCN, and three patients showed unilateral KCN. One of the KCN subjects had a history of keratoplasty in both eyes and was not included in the description of the refractive components in KCN subjects. Three KCN subjects had a history of corneal cross-linking in both eyes. Demographic and clinical descriptions of the KCN and non-KCN subjects are provided in Table 1. Figure 1 shows the distribution of refractive errors in the right and left eyes of the KCN subjects. In the KCN subjects, the steepest keratometry in the right eye was 48.66 ± 3.50 D, and the central corneal thickness was 475 ± 34 μm.

**Table 1:** Demographic and clinical distributions of characterizes in employees of Shiraz University of Medical Sciences

	Total population (mean ± SD) N = 2546 (1161 m/ 1385 f)	Non-keratoconus (mean ± SD) N = 25 (12 m/ 13 f)	Keratoconus (mean ± SD) N = 2521 (1149 m/ 1372 f)	P-value
Education level	Postgraduate 61.5% License 28.5% Diploma 10%	Postgraduate 80% License 16% Diploma 4%	Postgraduate 61.5% License 28.5% Diploma 10%	-
Age (year)	40.35 ± 6.70	39.32 ± 6.35	40.36 ± 6.71	0.43
Spherical Equivalent (D)	-0.75 ± 1.50	-0.70 ± 1.45	-4.04 ± 3.12	< 0.001
BCVA (logMAR)	0.01 ± 0.07	0.01 ± 0.07	0.06 ± 0.11	< 0.001
BMI (kg/m^2^)	26.81 ± 6.54	25.54 ± 4.16	26.82 ± 6.56	0.13
Glucose mg/dL	92.97 ± 17.71	92.17 ± 18.52	92.98 ± 17.71	0.53
LDL mg/dL	99.84 ± 23.54	110.48 ± 19.53	99.73 ± 23.55	0.01
HDL mg/dL	49.82 ± 10.08	51.83 ± 11.22	49.80 ± 10.07	0.58
Triglyceride mg/dL	136.36 ± 81.70	119.54 ± 55.82	136.52 ± 81.90	0.40
Maximum keratometry (D)	-	-	48.66 ± 3.50	-
Central corneal thickness (µm)	-	-	475 ± 34	-

(N = Number, m = male, f = female, BCVA = Best corrected visual acuity, BMI = body mass index, LDL = low density lipoprotein cholesterol, HDL = low density lipoprotein cholesterol)

In binary logistic regression analysis, age, sex, education level, and family history of KCN, BMI ≥ 30 kg/m^2^, LDL ≥ 110 mg/dL, HDL ≤ 40 mg/dL, tri-glyceride ≥ 150 mg/dL, and glucose ≥ 100 mg/dL were included. Family history of KCN was a significant risk factor (P < 0.001) with an odd ratio of 21.00 (95% CI: 9.00, 48.00). The probability of KCN in individuals with a family history of KCN is 21.00 times more likely than in individuals without a family history. LDL ≥ 110 mg/dL is a significant risk factor (P < 0.01) with an odd ratio of 3.00 (95% CI: 1.20, 6.40). The probability of KCN in individuals with LDL ≥ 110 mg/dL is 3.00 times more likely than in individuals with LDL < 110 mg/dL. The results of univariate and multivariate binary logistic regression analysis are provided in Table 2.

**Table 2:** Univariate and multivariable regression analysis for the potential risk factors of keratoconus

	Univariate Regression			Multivariable Regression		
	Odds Ratio	95% confidence interval	P-value	Odds Ratio	95% confidence interval	P-value
Age	0.97	(0.92, 1.04)	0.44	-	-	-
Sex	0.91	(0.41, 2.00)	0.81	-	-	-
Education level	1.37	(0.15, 12.38)	0.25	-	-	-
Family history of keratoconus	23.62	(10.40, 53.61)	0.00	21.00	(9.00, 48.00)	P < 0.001
BMI ≥ 30 mg/dL	0.44	(0.10, 1.10)	0.27	-	-	-
Glucose ≥ 100 mg/dL	0.41	(1.00, 1.77)	0.23	-	-	-
LDL ≥ 110 mg/dL	2.75	(0.51, 4.43)	0.01	3.00	(1.20, 6.40)	0.01
HDL ≤ 40 mg/dL	0.24	(0.03, 1.75)	0.16	0.40	(0.10, 3.00)	0.36
Triglyceride ≥ 150 mg/dL	0.40	(0.13, 1.14)	0.09	0.40	(0.13, 1.20)	0.10

(BMI = body mass index, LDL = low density lipoprotein cholesterol, HDL = low density lipoprotein cholesterol)

## Discussion

In the current study, the prevalence of KCN was 0.98% (95% CI, 0.6%-1.4%). This result is comparable with the results of Hashemi et al. in the urban area of northern Iran, where the prevalence of KCN was reported as 0.76% [36]. It is also comparable with the study by Armstrong et al. in the United Arab Emirates, where the prevalence of KCN was reported as 1.5% [37]. However, the prevalence of KCN in rural areas of Iran was reported as 3.3% [5], which is 3.3 times higher than our finding in Shiraz University of Medical Sciences Employees. The higher prevalence of KCN in rural areas of Iran was related to familial aggregation in rural regions of Iran. Also, the prevalence of KCN among university students and faculty members in Turkey is reported at 2.5%, which is higher than our finding; however, these results are within the limit of the confidence interval of the current study [35]. The lowest prevalence of KCN was reported in a population-based study in Denmark (which was 0.4%) [4], and the highest population-based study was in a rural area of Iran (3.3%) [5]. One of the important factors that can be attributed to differences in KCN prevalence is the measurement method. The current study applied retinoscopy and clinical examinations to detect KCN. Our study’s results are comparable to previous studies that used topography and clinical examination to detect KCN [33, 34]. Though, it is lower than the results of studies that used topography and the thinnest corneal point to detect KCN [35–37]. In addition, socio-economic factors and the imposed burden of KCN on social roles [38, 39] may also be attributed to our sample’s lower prevalence of KCN. University jobs are demanding and require higher education levels. Since KCN imposes burdens on near vision and role limitations [38, 39] some KCN patients may have chosen to avoid getting a role in these jobs and may have been omitted from our study subjects. As expected, the BCVA in KCN subjects was worse than the others (0.06 logMAR versus 0.00 logMAR). However, according to the definition of visual impairment, which is binocular visual acuity worse than 0.5 logMAR [25], KCN was not considered a risk factor for visual impairment in these subjects. Data analysis showed that the probability of visual impairment in the KCN group was zero. Whereas Hashemi et al. reported that the likelihood of visual impairment in KCN subjects is eight times greater than that in healthy individuals [33]. This difference could be attributed to the study population. The current study population included educated people with sufficient access to treatment options. There was 1 case (4% in KCN subjects) underwent successful keratoplasty in both eyes with the BCVA of 0.1 logMAR and three patients (12% in KCN subjects) who underwent corneal crosslinking. Current treatment modalities, such as corneal crosslinking, keratoplasty, and contact lenses, can lead to more successful management of KCN and decrease the risk of visual impairment in KCN subjects.

In the current study, we evaluated the associations between family history of KCN age, sex, and education levels. We found that family history was the most prominent risk factor for developing KCN, suggesting a strong genetic trait in KCN. Our results showed that the probability of KCN incidence in individuals with a positive family history was 20 times higher than in patients with a negative family history. This finding is similar to that of Wang Y et al. [40], who estimated that the relative of an individual with KCN has a 15 to 67 times greater risk of developing KCN than an individual with no family history of KCN. Our data analysis showed a similar prevalence of KCN in male and female subjects, suggesting that KCN is sex-independent. This finding is similar to previous population-based studies [33, 37, 41]. Genetic investigations have revealed autosomal chromosomes involved in the KCN disease [42] and no evidence for an x-linked genetic pattern has been revealed until now. However, some studies reported a higher prevalence in the male population than in females [3, 5, 43] relating it to the higher pressure of eyelids in males than in females [3]. The current study also showed that the prevalence of KCN is independent of age. The mean age in the present study was 39.32 ± 6.35 years at the time that almost all of the KCN cases were manifested. More recent population-based studies have reported a higher prevalence of KCN than earlier studies. [44, 45] It is thought that the prevalence of KCN has increased in the younger generation. One reason that KCN was not associated with age in this report is that university employees are mostly of similar age status. Although the age range is wide in the current study, most employees are of similar and comparable age. However, this effect can also be attributed to the development of tomographical and topographical devices in the recent years that precisely detect KCN. [3, 46].

Also, the association between KCN and popular biomarkers of oxidative stress that are known in metabolic disorders such as BMI ≥ 30 kg/m2, LDL ≥ 110 mg/dL, HDL ≤ 40 mg/dL, triglyceride ≥ 150 mg/dL, and glucose ≥ 100 mg/dL, were evaluated in the current study. The data analysis showed that elevated serum level of LDL in the blood contributes as a risk factor for KCN disease. We found that the risk of KCN in individuals with a serum level of LDL ≥ 110 mg/dL is three times (95% CI: 1.20, 6.40) greater than in individuals with a serum level of LDL < 110 mg/dL. Previous studies revealed associations between lipids and KCN [12, 13, 22]. A pilot molecular study showed that metabolic pathways involved in energy production, lipid metabolism, and amino acid metabolism differ between KCN corneas and normal corneas [22]. On the other hand, elevated levels of inflammatory biomarkers, including monocyte-to-HDL-cholesterol ratio and neutrophil-to-lymphocyte ratio, in KCN patients have been reported in previous studies suggesting an inflammatory background in KCN [12, 13, 22, 47]. Since a high serum level of LDL increases the risk of oxidative stress [48] and oxidized LDL triggers inflammatory reactions [47, 48], the current finding adds evidence for an inflammatory background in KCN. Moreover, elevated serum ferritin levels are associated with liver fat and inflammation [49, 50]. Deposits of ferritin in the corneal basal epithelial cells are a known sign of Fleischer’s ring in some patients with KCN [51, 52]. Therefore, there might be a link between the up-regulation of LDL and ferritin deposits in patients with KCN. The current study suggests that more investigation into the inflammatory background in KCN disease is required. These investigations may open new areas for medication treatment and diet suggestions for KCN patients.

The study’s strength is the evaluation of lipid profiles in KCNs. In the current study, the Pentacam imaging was obtained only for suspected KCNs according to clinical examinations, and it was not performed for all participants, which was a study limitation. We might not identify some forme fruste and mild KCNs; however, our prevalence result and the confidence interval are comparable with the previous studies. Another limitation was the study target which was university employees. From the public health viewpoint, a population-based study is recommended; however, the current study participants lived in diverse city locations with different education levels and socioeconomic backgrounds.

## Conclusions

In conclusion, we found a prevalence of 0.98% (CI, 0.6%-1.4%) for KCN disease among the Shiraz University of Medical Science employees. The prevalence of KCN was not related to education level, and the risk of visual impairment is not associated with KCN in educated individuals. Family history is a known risk factor for KCN and was confirmed in the present study. We also found elevated serum levels of LDL in the blood to be a risk factor for KCN in the study subjects.

## Data Availability

The datasets used and/or analysed during the current study are available from the corresponding author on reasonable request.
